# Dihydro-5,6-dehydrokavain (DDK) from *Alpinia zerumbet*: Its Isolation, Synthesis, and Characterization

**DOI:** 10.3390/molecules200916306

**Published:** 2015-09-09

**Authors:** Tran Dang Xuan, Rolf Teschke

**Affiliations:** 1 Division of Development Technology, Graduate School for International Development and Cooperation (IDEC), Hiroshima University, Higashi Hiroshima 739-8529, Japan; E-Mail: tdxuan@hiroshima-u.ac.jp; 2 Department of Internal Medicine II, Division of Gastroenterology and Hepatology, Klinikum Hanau, Academic Teaching Hospital of the Medical Faculty of the Goethe University, Frankfurt/Main, 63450 Hanau, Germany

**Keywords:** DDK, dihydro-5,6-dehydrokavain, dehydrokavain, kavalactones, shell ginger, *Alpinia zerumbet*, *Piper methysticum*

## Abstract

Dihydro-5,6-dehydrokavain (DDK) is the major and most promising component of the tropical plant *Alpinia zerumbet* (shell ginger), a species of the ginger family Zingiberaceae. *Alpinia zerumbet* is known for its human use as a traditional herbal medicine, food, and dietary supplement. With its α-lactone ring, DDK belongs to the large chemical group of kavalactones, which are also found in kava (*Piper methysticum*), another herbal medicine; DDK is characterized by a double-bond linkage at positions 5,6 and the absence of a double-bond linkage at positions 7,8. This dissociates DDK from other kavalactones with their linkages at positions 7,8 and 5,6 that are both either completely saturated or unsaturated, or may have an unsaturated bond at the position 7,8 as well as a saturated bond at the position 5,6. DDK is easily identified and quantified by HPLC and GC. DDK contents in fresh leaves, stems and rhizomes range from 80 to 410 mg/g, requiring solvent extraction procedures to ensure high DDK yield. This is best achieved by hexane extraction from fresh rhizomes that were previously boiled in water, allowing DDK yields of up to 424 mg/g. Successful synthesis of DDK can be achieved by asymmetric pathways, whereas its simple chemical structure facilitates the synthesis of DDK derivatives by HCl hydrolysis. Thus, all synthesized products may be used for various commercial purposes, including the potential development of promising antiobesity pharmaceutical drugs, preparation of specific and safe dietary supplements, and use as effective natural herbicides or fungicides.

## 1. Introduction

Dihydro-5,6-dehydrokavain (DDK) is the major constituent of *Alpinia zerumbet*, a plant widely growing in the tropics [1] that represents a species of the *Alpinia* group [2]. *Alpinia* is the largest genus of the family Zingiberaceae, classified by Charles Plumier, and is named after Prospero Alpino, the well-known Italian botanist of the sixteenth century. Plants of the genus *Alpinia* and their constituents have numerous positive effects, including antimicrobial, antiparasitic, insecticidal, anticancer, antiproliferative, antiinflammatory, analgesic, antiallergic, neuroprotective, and antioxidant properties [2]. Some plants of the *Alpinia* genus also may exert specific beneficial effects, related to osteoarthritis [3], aging [4], gastric cancer [5], and diabetes [6]. Similar to these positive features [2,3,4,5,6] are the promising properties specifically described for *Alpinia zerumbet* and its anti-obesity effects elicited by DDK and 5,6-dehydrokawain (DK) [6,7] including its derivative hispidin that can be obtained by hydrolysis in stomach acid and subsequent metabolism by hepatic microsomal CYP2C9 [8]. All three chemicals increased lipolysis when incubated with differentiated 3T3-L1 adipocytes, using glycerol release as parameter. Compared to controls, hispidin, DDK, or DK significantly increased glycerol release by 276%, 225%, or 137%. All these compounds also reduced intracellular triglycerides in a dose-dependent manner [7]. Therefore, *Alpinia zerumbet* with its ingredients and hispidin may well establish a new therapeutic principle for anti-obesity with lipocytes as the primary organ target rather than liver or intestinal cells.

Most of these properties have been reported in experimental studies and not yet in humans [1,2,3,4,5,6]. Nevertheless, it appears that plants of the *Alpinia* genus have some potential of further research and development. Others have even called *Alpinia* the gold mine of future therapies [2]. Whether or not this perspective also applies to *Alpinia zerumbet* syn. *Alpinia speciosa* remains to be established by further research and clinical trials, proving therapeutic efficacy, lack of major adverse reactions, and establishing a positive benefit/risk profile. These next steps will be challenging and require the expertise of clinicians familiar with the criteria of evidence based medicine, focusing on those diseases that best profit from a tentative treatment by *Alpinia zerumbet*.

The present review article provides detailed information of current developments and trends in research of *Alpinia zerumbet* with focus on DDK, its extraction, properties, and synthesis. For reasons of comparison, DDK merits additional but only brief attention, as this chemical is a component also of another plant, namely kava (*Piper methysticum*). Water-based herbal extracts derived from kava rhizomes exert anxiolytic properties [9,10,11,12,13] and are known for their therapeutic use after proven efficacy by evidence based medicine criteria [13]. Questions of therapeutic efficacy in selected ailments also pertain to *Alpinia zerumbet*, the key issue will be how to achieve clinical trials to establish efficacy in few selected ailments. Based on present knowledge, a systematic approach should also be feasible to further characterize *A. zerumbet* with its DDK.

## 2. Chemical Structure of DDK

**Figure 1:**
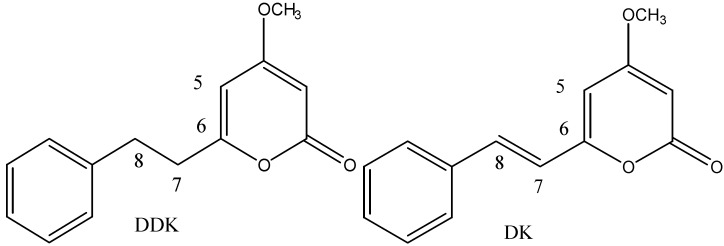
Chemical structure of the kavalactones DDK and its derivative DK isolated from *Alpinia zerumbet* (shell ginger) and from *Piper methysticum* (kava).

According to their pyrone moieties, kavalactones can be classified as types A–D through the presence or absence of double-bond linkages at positions 5,6 and 7,8. Type A is characterized by the absence of double-bond linkages in both positions 5,6 and 7,8. Conversely, type B has an unsaturated bond at position 7,8, and type C is completely unsaturated at both positions [9]. Type D has a saturated bond at position 7,8 but position 5,6 is unsaturated. Therefore, DDK belongs to the type D, its derivative DK to type C (Figure 1). Structurally, the biological activities of kavalactones depend on differences of linkages at positions 5,6 and 7,8 [9]; this likely explains the various biological activities of the individual kavalactones including DDK and DK present in *Alpinia zerumbet* and kava, but further studies are needed.

## 3. Isolation and Identification of DDK

### 3.1. DDK Isolation from Alpinia zerumbet

DDK can be isolated from fresh rhizomes of *Alpinia zerumbet* by extraction with methanol [14]. This methanol extract is shaken with petrol and then with chloroform. From the chloroform soluble fraction, DDK may be separated by silica gel chromatography. As an alternative applied to *Alpinia zerumbet* leaves, extraction with acetone requiring one month was described [15]. An essential oil fraction is obtained by steam distillation of the extract. Its non-volatile fraction is extracted with *n*-hexane, benzene, ethyl acetate, and then *n*-butanol. The *n*-hexane and benzene extracts were fractionated by silica gel column chromatography to yield DDK.

Although these methods lead to a successful isolation of DDK, the overall isolation procedures remain complex and time-consuming. Subsequently, a more practicable and simplified method to isolate DDK was described by soaking leaves of *Alpinia zerumbet* in boiling water for 2–3 h [15]. These water based crude extracts can be further processed by extraction using chloroform as solvent to yield DDK, but traces of impurities consisting of essential oils and DK commonly remain. A yellowish solid material is obtained by evaporating the chloroform layer, and the resulting product again is dissolved in boiling water, followed by rapidly filtering out the insoluble matter [15]. The resulting filtrate is crystallized at 8 °C to yield DDK. This isolation method for DDK is superior to other reported methods because column chromatography is not any more necessary, but it is hampered by the use of chloroform with its known toxicity risk. In our laboratory, hexane replaces now the chloroform as solvent to reduce the inhalative risk of chloroform toxicity. This is associated with a higher yield of DDK compared to chloroform [9].

### 3.2. DDK Isolation from Kava

Kava contains at least 18 kavalactones and many other compounds, rendering the isolation of DDK from its extracts much more complex [9] than its simple isolation from the hexane layer of *Alpinia zerumbet* [16,17,18]. Column chromatography with acetone, chloroform, and water is effective to separate DDK and major kavalactones from kava roots [9]. Other solvents such as methanol, ethanol, and hexane are less effective [14]. Protocols for identification and isolation of DDK, using HPLC, GC-MS, LC-MS, TLC, and CC, were established [15,16,17,18,19]. The effectiveness of different extracting solvents regarding DDK yields was also examined [9].

### 3.3. Identification

**Figure 2:**
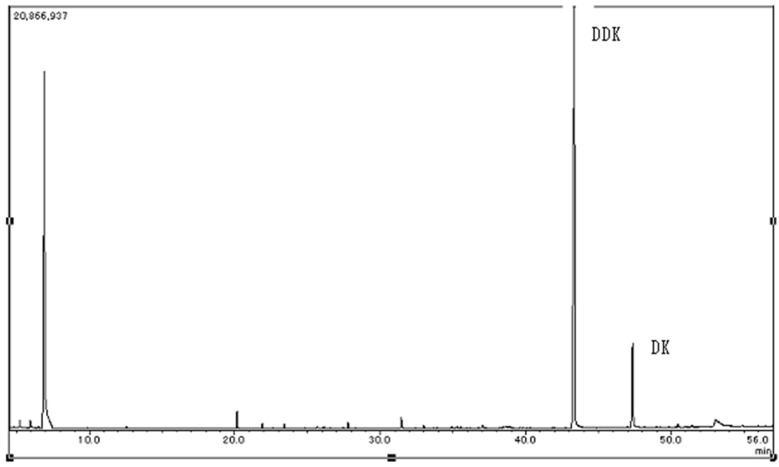
Detection of DDK and DK by GC-MS, obtained from hexane extract derived from leaves of *Alpinia zerumbet* [17,18].

DDK can be identified by GC-MS (QP-2010, Shimadzu Co., Kyoto, Japan) [17,18]. DDK and DK were detected at 43.3 and 47.3 min, respectively. Other peaks were unknown (Figure 2).

The isolation of DDK was first reported 40 years ago from *Alpinia zerumbet* rhizomes [20], and its structure of dihydro-5,6-dehydrokavain was assessed by spectroscopic methods of IR, NMR, and mass spectrum [8,14,20]. The presence of DDK in *Alpinia zerumbet* leaves was later confirmed, using ^1^H-NMR, ^13^C-NMR, IR, FDMS and EIMS [9]. DDK was also found in other plant parts of *Alpinia zerumbet* [15,16,17,18,21]. LC-MS is also efficient to determine DDK but GC-MS is much convenient as DDK is not completely dissolved in LC solvents such as water and methanol.

## 4. Efficacy of Extraction Solvents

As the structure of DDK looks hydrophobic, several organic extracting solvents such as methanol and acetone have been applied to isolate DDK from *Alpinia zerumbet* [8,14,20], but the yields were low and did in no way reflect the actual DDK amounts present in the plant. To increase the yield and efficacy of isolation, the leaves or rhizomes of *A. zerumbet* were boiled in water for 2–3 h, and after cooling and filtering, the water extract was then subjected to various organic solvents as the preferred method [15]. Actually, the melting point of DDK is 96–97 °C, enhancing the release of large amounts of DDK from Alpinia plant parts and reaching DDK yields of up to 424 mg/g and 148 mg/g from fresh rhizomes and leaves, respectively [18]. These figures were higher compared to previous research when fresh plant parts of *Alpinia zerumbet* were cut and soaked by ethanol for one week, which resulted in lower DDK amounts of 350 mg/g in fresh rhizomes but with a high purity (>95%) of DDK [15]. Among the boiled solvents, chloroform, hexane, methanol, ethanol, and acetone had been examined [9]. Chloroform and hexane are the most effective solvents to yield large amounts of DDK; under toxicity aspects, hexane is now the preferred solvent over chloroform.

## 5. Quantification of DDK

### 5.1. Approaches

DDK can be quantified by HPLC as described earlier [15], with some modifications such as the use of gradient and column as described below to improve the accuracy of DDK quantification [17,18]. DDK was detected at 35 min by comparing the retention time of the authentic compound.

### 5.2. Content of DDK in Alpinia zerumbet

DDK and DK as well as multiple other chemicals are found in *Alpinia zerumbet* (Table 1). Generally, all plant parts of *Alpinia zerumbet* including leaves, stems, rhizomes, flowers, and seeds contain both DDK [15,19] and its derivative 5,6-dehydrokavain (DK, Figure 1) [14,18,19]. Other compounds of relevance include phenolics, essential oils, labdadienes, catechins, kaempferol, zerumins, chalcones, flavanones, chalcones, cardamonin, alpinetin, and rutin (Table 1) [17,18,19,20,21,22,23,24,25,26,27,28,29,30,31,32]. Major bioactive chemicals are in the leaves, but some are found in rhizomes and seeds (Table 1). Essential oils from leaves and rhizome have main components of 1,8-cineol, camphor and methyl cinnamate [17,18,19]. At an industrial level, essential oils of *Alpinia zerumbet* are obtained by steam distillation of its leaves. On a quantitative basis, the yields of essential oils as the preferred products are low. From about 100 kg fresh leaves of *Alpinia zerumbet*, 100 L of distillate can be obtained, from which at best 100 mL of essential oils can be produced. In Japan with preference of Okinawa, oils of *Alpinia zerumbet* are used as cosmetics, perfumes, and soaps [19]. Isothymol, thymol, and eugenol in the essential oils of *Alpinia zerumbet* possess strong antifungal activity against plant pathogenic fungi [19]. Antioxidant and antibacterial activities of DDK, essential oils, and phenolics in alpinia were investigated, which were also sprayed with copper sulphate [17]. After stressed by copper sulphate, DDK increased but essential oils decreased, suggesting that DDK plays some role in plant defense system of alpinia [17].

**Table 1:** DDK, DK, and other chemicals in various plant parts of *Alpinia zerumbet*.

Chemical Name	Plant Part	References
Dihydro-5,6-dehydrokawain (DDK)	Leaves, stems, rhizomes	[15]
5,6-Dehydrokawain (DK)	Leaves, stems, rhizomes	[15]
Essential oils	Leaves, roots	[17,18,19,20]
(*E*)-Labda-8(17)-12-diene-15-ol-16-al	Rhizomes	[21]
(*E*)-15,16-Bisnorlabda-8(17)-11-diene-13-one	Rhizomes	[21]
Phenolic acids	Leaves, stems, rhizomes	[14,17,18]
12-Labdaiene-15,16-dial (labdadiene)	Rhizomes	[22]
Rutin	Leaves	[23]
Kaempferol-3-*o*-glucuronide	Leaves	[23]
(+) Catechin	Leaves	[23]
(−) Epicatechin	Leaves	[23]
*p*-Hydroxycinnamalehyde	Rhizomes	[24]
[Di-(*p*-hydroxy-*cis*-styryl)] methane	Rhizomes	[24]
1,7-Diarylheptanoid	Rhizomes	[25]
Zerumin A	Seeds	[26]
Zerumin B	Seeds	[26]
Kaempferol-3-*o*-rutinoside	Leaves	[27]
Coronarin E	Seeds	[26,27]
(*E*)-15,16-Bisnorlabda-(8(17)-11-diene-13-one	Seeds	[28]
Dihydroflavokavain B	Rhizomes	[14]
Cardamonin (2′,4′-Dihydroxy-6′-methoxy chalcone)	Seeds	[28]
Alpinetin (7-hydroxy-5-methoxy flavanone)	Seeds	[28]
*Trans*-1-(4′-Hydroxy-3′-methoxyphenyl-7-phenylhept-1-*en*-3-one (yakuchinone-B)	Pericarps	[29]
1-(4′-hydroxy-3′-methoxyphenyl)-7-phenyl-3-heptanone	Fruits	[30]
Labda-8(17),12-diene-15,16-dial	Rhizomes	[31]
Bisabolanes	Leaves	[31]
Steroids	Seeds	[32]

On a quantitative basis, DDK and DK show differences in amounts in fresh leaves, stems, and rhizomes of *Alpinia zerumbet*, with higher amounts of DDK compared to DK in all plant parts (Table 2) [15]. Contents of DDK and essential oils in alpinia are varied among varieties, locations, and time within a year (unpublished laboratory data). The reason why alpinia accumulates such a large amount of DDK and DK in leaves and rhizomes is unknown. It can be proposed that DDK and DK may play an important role in allelopathy of alpinia to suppress growth of other plants in its vicinity and expands its population in the plant ecosystem.

**Table 2:** DDK and DK contents in plant parts of *Alpinia zerumbet* [15].

Compounds	Quantity (mg/g of Fresh Weight)		
	Leaves	Stems	Rhizomes
Dihydro-5,6-dehydrokawain (DDK)	410.0	80.0	350.0
5,6-Dehydrokawain (DK)	10.0	20.0	100.0

### 5.3. Content of DDK in Kava

In addition to *Alpinia zerumbet* (Table 1 and Table 2)*,* DDK is also found in kava (*Piper methysticum*) rhizomes (Table 3) as one of a total of 18 kavalactones [9]. Compounds other than DDK, DK, and kavalactones are flavokavains, phenolics, chalcones, pipermethystine, flavanones, and glutathione (Table 3) [9,33,34,35,36]. Except for DDK, DK, and other kavalactones, phenolic acids, and glutathione that were quantified, contents of other compounds such as chalcones, flavanones, cinnamic acid bornyl ester, 2,5,8-trimethyl-1-naphthol, 5-methyl-1-phenylhexen-3-yn-5-ol have not been determined in kava [9,33,34,35,36]. All compounds detected in kava were from rhizomes (Table 3), but their presence in leaves and stems have not been studied.

**Table 3:** DDK, DK, and other chemicals in various plant parts of kava.

Chemical Name	Plant Part	References
Phenolic acids	Rhizomes	[33]
Dihydro-5,6-dehydrokavain (DDK)	Rhizomes	[9]
5,6-Dehydrokavain (DK)	Rhizomes	[9]
Other kavalactones: dihydromethysticin, 7,8-dihydrokavain, kavain, methysticin, desmethoxyyangonin, yangonin, dihydro-5,6-dehydrokavain, methoxyyangonin, hydroxykavain, 7,8-dihydrokavain, 11-hydroxy-12-methoxydihydrokavain, 5,6,7,8-tetrahydroyangonin, 5,6-dehydromethysticin 11,12-dimethoxydihydrokavain, 11-methoxy-12-hydroxydehydrokavain, 11-hydroxyyangonin	Rhizomes	[9]
Flavokavain A	Rhizomes	[9]
Flavokavain B	Rhizomes	[9,34,35]
Flavokavain C	Rhizomes	[9]
Cinnamic acid bornyl ester	Rhizomes	[9]
5,7-Dimethoxyflavanone	Rhizomes	[9,35]
2,5,8-Trimethyl-1-naphthol	Rhizomes	[9]
5-Methyl-1-phenylhexen-3-yn-5-ol	Rhizomes	[9]
8,11-Octadecadienoic acid-methyl ester	Rhizomes	[9]
Pinostrobin chalcone	Rhizomes	[9,35]
5-hydroxy-4′-7-Dimethoxyflavanone	Rhizomes	[9]
5,7(OH)2-4′-one-6,8-dimethylflavone	Rhizomes	[9]
Pipermethystine	Rhizomes	[9,36]
Glutathione	Rhizomes	[9]

The yields of DDK show a wide range, depending on the type of the extracting medium: hexane, ethanol, methanol, chloroform, acetone, or water (Table 4) [9]. Among the seven major kavalactones, there are four different categories, types A-D [9]: to type A belong the kavalactones dihydromethysticin and 7,8-dihydrokavain; to type B kavain and methysticin; to type C desmethoxyyangonin and yangonin; and to type D dihdydro-5,6-dehydrokavain (Table 4). In kava roots and on a quantitative basis, kavalactone type A predominates with variable results for the other types; among the examined extracting solvents, acetone is the most effective one, followed by water and chloroform (Table 4). The extraction by water is preferred as it is safer than chloroform.

**Table 4:** Content of DDK and other major kavalactones in different extracting solvents prepared from kava roots (mg/g extract) [9]. * Structure of kavalactones divided by types A–D moieties.

Kavalactones	Moieties *	Quantity (mg/g Extracting Solvents)					
		Water	Acetone	Chloroform	Methanol	Ethanol	Hexane
Dihydromethysticin	A	31.5	51.9	18.9	5.4	3.2	3.6
7,8-Dihydrokavain	A	3.8	55.1	23.0	18.6	9.4	10.1
Kavain	B	36.9	41.5	14.7	6.9	3.3	4.7
Methysticin	B	0.0	5.5	14.4	0.0	0.0	1.2
Desmethoxyyangonin	C	6.7	21.0	7.6	4.3	2.1	2.7
Yangonin	C	6.8	84.1	22.9	5.7	2.4	2.1
Dihydro-5,6-dehydrokavain	D	22.9	27.1	4.7	4.7	2.1	1.9
Total kavalactones		108.6	286.2	106.2	45.6	22.5	26.3

## 6. DDK in Other Alpinia Species

Kavalactones have been identified in other Alpinia species such as *Alpinia kumatake* [37], *Alpinia galanga* [38,39], and *Alpinia oxyphyllae* [38,39,40,41]. However, DDK and DK coexist only in *Alpinia zerumbet* [18,19] and *Alpinia kumatake* [37], and not in *Alpinia galangal* and *Alpinia oxyphyllae* [39,40]; DDK is also present in *A. blepharocalyx* but it was not quantified [41]. Interestingly, *P. methysticum*, *A. zerumbet*, and *A. belepharocalyx* all contain DDK as major biological constituent, although they belong to two different plant families, namely the Piperaceae and the Zingiberaceae family, respectively.

To date, there is to our knowledge not a single report investigating whether kavalactones other than DDK and DK are present in *Alpinia zerumbet* and other *Alpinia* species. Therefore, further studies are required to evaluate whether other kavalactones are found at least in minimal amounts just above the detection limit or whether there is evidence of complete lack of these additional kavalactone compounds. Overall, this may be an interesting detail to further elucidate the biosynthesis of other kavalactones; additional experimental studies should focus on the various enzymatic pathways leading to the synthesis of kavalactones, comparing *Piper methysticum* and *Alpinia* and studying the genetic relevance of variable kavalactone synthesis of these two plant species. The high number of different kavalactones synthesized by kava is a specific phenomenon.

## 7. Synthesis of DDK and DDK Derivatives

**Scheme 1:**
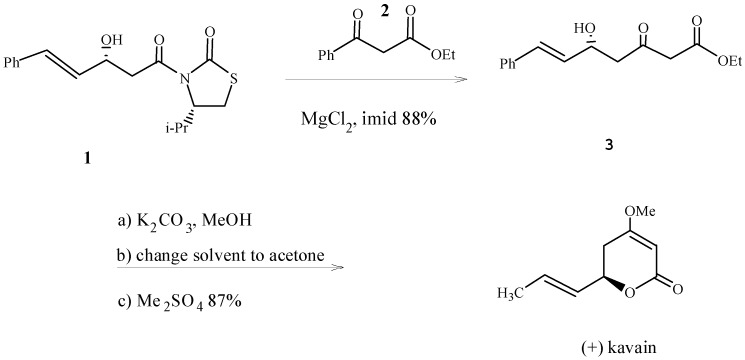
Three asymmetric pathways to synthesize kavalactones [42]. **1**: *N*-acetyl- thiazolidinethione; **2**: Potassium salt of monoethyl malonate; **3**: Phenyl-δ-hydroxy-β-ketoester.

Three asymmetric pathways to synthesize kavalactones are described [42], which also represented the first synthesis of kavain. The first method is chiral auxiliary-based and utilizes aldol reactions of *N*-acetyl thiazolidinethiones, followed by a malonate displacement/decarboxylation reaction. The second approach uses the asymmetric catalytic additions [43] of dienolate nucleophile equivalents developed earlier [44,45]. The third approach uses tin-substituted intermediates, as advanced general precursors of kavalactone derivatives via Pd(0)-catalyzed Stille couplings [46] with aryl halides (Scheme 1). These are the three most simple and efficient approaches to the asymmetric synthesis of the kavalactones, which is acknowledged as the first enantioselective synthesis of (+)-kavain [42].

**Figure 3:**
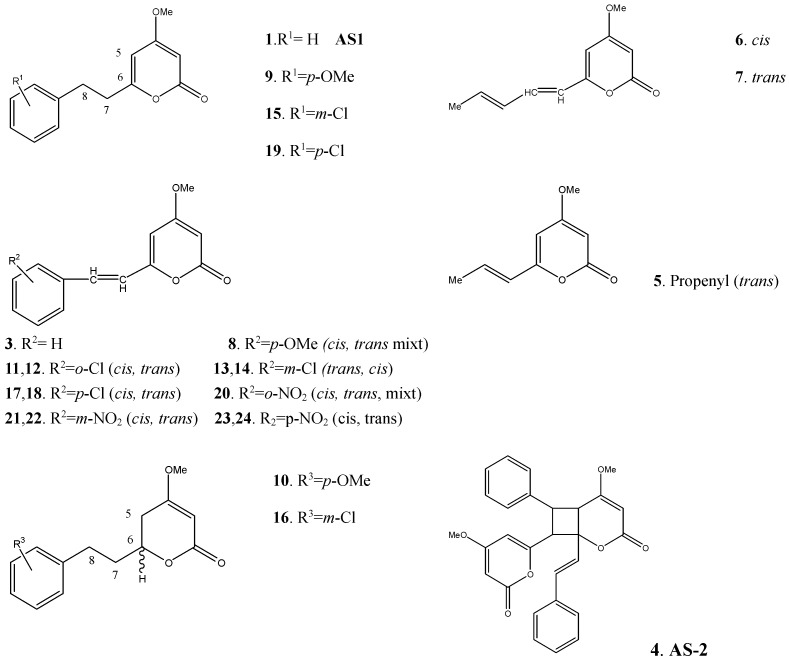
Some DDK derivatives synthesized by Fujita *et al.* [16].

DDK and several DDK derivatives have been synthesized (Figure 3) [16]. The compounds **AS-1** and **AS-2** were separated from *Alpinia zerumbet* leaves, extracted with Me_2_CO for a month and extracted with *n*-hexane and benzene to isolate the **AS-1** as a plant growth inhibitor, **AS-2** was a minor compound. The DDK was synthesized by conversion of 4-methoxy-6-methyl-2*H*-pyran-2-one (**2**) to 4-methoxy-6-styryl-2*H*-pyran-2-one (**3**). Then this was converted to the 6-styryl derivative. The preparation of DDK derivatives was carried out in a similar manner to that of DDK, using the corresponding triphenylphosphonium and PtO_2_ as a catalyst, hydrogenating the 6-(*p*-methoxystyryl) and 6-(*m*-chlorostyryl) derivatives to yield DDK derivatives (Figure 3).

**Scheme 2:**
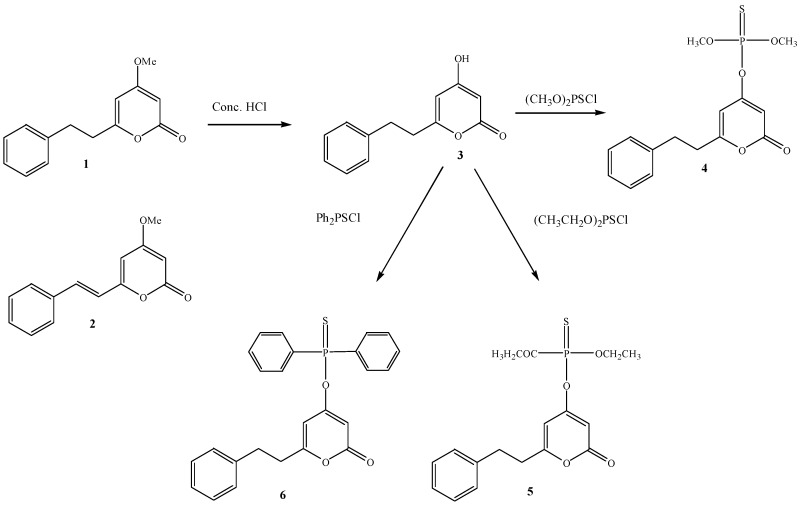
Structures and synthesis route of DDK derivatives [15]. **1**: DDK; **2**: DK; **3**: 4-Hydroxy-6-(2-phenylethyl)-2*H*-pyran-2-one; **4**: Dimethyl-[6-2-phenyethyl]-2-oxo-2*H*-pyran-4-yl]-phosphorothionate; **5**: Diethyl-[6-(2-phenylethyl)-2-oxo-2*H*-pyran-4-yl] phosphorothionate; **6**: [6-(2-Phenylethyl)-2-oxo-2*H*-pyran-4-yl]-diphenylphosphorothionate.

Tawata *et al.* [15] synthesized some DDK derivatives as shown in Scheme 2. DDK was hydrolyzed by HCl to yield hydroxy-6-(2-phenylethyl)-2*H*-pyran-2-one which has a hydroxyl group instead of a methoxy group at position 4 of DDK. Compound **3** was reacted with dimethyl chlorothiophophate, diethyl chlorothiophosphate, and diphenylphosphinothioyl chloride by using trimethylamine as a base to yield three derivatives **4**, **5** and **6**. The IR spectra of the compounds showed the existence of P=S, P-O, and P-O-C groups. ^1^H-NMR and ^13^C-NMR data showed similar chemical shifts in the values for the 6-(2-phenylethyl)-2-oxo-1*H*-pyran-4-yl group.

## 8. Perspectives

Molecular aspects of DDK and its derivatives have thoroughly been examined, awaiting pragmatic approaches of their further usage as drugs, dietary supplements, and agriculture products. Based on DDK, its derivatives, and other ingredients, *Alpinia zerumbet* is a promising plant in analogy of other alpinia species with known positive properties, which all belong to the well researched Zingiberaceae family. These plants are edible and may provide little risks for human use, characteristics which merit further studies of possible applications.

## 9. Conclusions

DDK can easily be obtained by synthesis or isolation from *Alpinia zerumbet*. It is synthetized very conveniently by asymmetric pathways, whereas its simple chemical structure facilitates the synthesis of DDK derivatives by HCl hydrolysis. In addition, DDK and its derivatives are easily isolated by solvents such as chloroform and hexane, preferentially using rhizomes of *Alpinia zerumbet* to obtain a high yield of the compounds. All synthesized products as well as *Alpinia zerumbet* itself appear promising, awaiting further proof of efficacy and safety. They may be used for various commercial purposes, including the potential development of future pharmaceutical drugs, preparation of specific and safe dietary supplements, and use as effective natural herbicides or pesticides. It appears that the successful exploitation of this ginger plant also may help to improve the rural development in the tropics.
